# A systematic review and meta-analysis of integrated traditional Chinese medicine and Western medicine in treating glomerulosclerosis

**DOI:** 10.1097/MD.0000000000024799

**Published:** 2021-02-19

**Authors:** Yue-tong Wang, Rong-qiang Zhang, Shu-fei Wang, Xian-cheng Li, Nan Zhang, Ya-feng Zhao, Yu Wang, Xiao-yong Yu, Kai Qu

**Affiliations:** a School of Basic Medicine; b School of Public Health, Shaanxi University of Chinese Medicine, Xianyang; c Nephrology Department, Shaanxi Traditional Chinese Medicine Hospital, Xi’an, Shaanxi, China.

**Keywords:** glomerulosclerosis, meta-analysis, traditional Chinese medicine and Western medicine (TCM+WM)

## Abstract

**Background:**

The combination of Traditional Chinese medicine and Western medicine (TCM+WM) has been widely used in the treatment of glomerulosclerosis, but the results are still controversial. This study will assess the clinical efficacy of TCM+WM for glomerulosclerosis and provide evidence-based medical data via meta-analysis.

**Method:**

The MEDLINE, EMBASE, PubMed, Cochrane Central Registry of Controlled Trials, and multiple Chinese databases (Wan Fang, CNKI, and VIP) were searched for randomized controlled trials (RCT) that compared the effects of WM and TCM+WM. Review Manager 5.3 software was used for the meta-analysis of selected studies, and appropriate tests were performed to determine the quality, heterogeneity and sensitivity of these studies.

**Results:**

Sixteen RCTs met the inclusion criteria and were selected for the analysis. Compared with the placebo or WM-treated glomerulosclerosis patients, TCM+WM intervention significantly improved renal function indices including 24-hour urine protein quantity (24 h U-Pro), serum creatinine (Scr), blood urea nitrogen (BUN), creatinine clearance (Ccr). In addition, the serum albumin (ALB), triglyceride (TG), and cholesterol (CHOL) levels were also significantly improved (*P* < .05) in patients receiving the combination therapy. Finally, the combination of TCM+WM reduced the indices of glomerulosclerosis more effectively compared with WM alone.

**Conclusion:**

The combination of TCM+WM can significantly improve the renal function and prognosis of patients with glomerulosclerosis.

## 1 Introduction

Glomerulosclerosis is the primary pathological basis for the progression of chronic kidney disease (CKD) to end-stage renal disease (ESRD),^[1]^ and the direct cause of 25.8% of the ESRD cases.^[2]^ Although the incidence rate varies depending on the race, sex, age, primary disease etc, it places a considerable socio-economic burden on the patients. The most common symptom of glomerulosclerosis is proteinuria, along with hematuria, hypertension, renal insufficiency, etc.^[3]^ It is currently treated with hormones, angiotensin converting enzyme inhibitor (ACEI), and immunosuppressants,^[4]^ which can be supplemented with lipid-lowering, anticoagulation, and hypotensive drugs. Nevertheless, the high recurrence rate and adverse reactions have greatly limited the outcomes of these strategies.^[5,6]^ Traditional Chinese medicine classifies glomerulosclerosis as “consumptive disease,” “urine turbid” etc, and the herbal preparations have been very effective in mitigating the symptoms.^[7,8]^ We conducted a systematic review and meta-analysis of randomized controlled trials (RCTs) that compared the therapeutic effects of Western medicine (WM) and TCM+WM on patients with glomerulosclerosis.

## 2 Methods

The Cochrane Handbook for Systematic Review of Interventions and Preferred Reporting Items for Systematic Reviews and Meta-Analysis (PRISMA)^[9]^ were followed for all steps.

### 2.1 Search strategy

The Cochrane library, EMBASE, PubMed and MEDLINE databases, and Chinese language databases including VIP, Wan Fang, and CNKI were searched for relevant RCTs published from June 2001 to November 2019. The following keywords were used to search the English-language databases: “traditional Chinese medicine,” “TCM,” “glomerulosclerosis,” “Western medicine,” “combination,” “RCT,” “Formulas of Chinese medicine,” and “clinical trials.” The Chinese databases were searched using the following keywords:

“Shen Xiao Qiu Ying Hua,” “Zhong Xi Yi Jie He,” “Fang,” “Lian He,” “Sui Ji Dui Zhao Shi Yan,” and “Lin Chuang Yan Jiu.” The retrieved papers were screened by 2 authors based on the title and abstract, and the bibliography of the selected papers was further screened manually to identify additional RCTs. In case of any issues with the trial design or results or other ambiguities, the corresponding authors were contacted for clarification. Ethical approval was not necessary since animal models or human subjects were not involved.

### 2.2 Inclusion and exclusion criteria

The studies were selected based on the following inclusion criteria: confirmed diagnosis of glomerulosclerosis, RCT design, comparison of WM-treated (control) and TCM+WM-treated (treatment group) patients, minimum treatment duration of 4 weeks, evaluation of renal function (24-hour urine protein quantity [24 h U-Pro], serum creatinine [Scr], blood urea nitrogen [BUN], creatinine clearance [Ccr]), serological (serum albumin [ALB]), and metabolic (triglyceride [TG], cholesterol [CHOL]) indices. Studies with unclear diagnostic criteria of glomerulosclerosis, non-RCT design, inclusion of other treatment strategies, retrospective design, literature reviews, and inaccurate/incomplete data were excluded.

### 2.3 Study selection and quality assessment

After excluding the irrelevant papers, 2 reviewers independently screened the RCTs according to the established inclusion criteria. The results were compared and any differences are resolved through discussion or a third reviewer. The Jadad scale was used for quality assessment based on randomization, blinding, controlled, withdrawals, and dropouts.^[10]^ Studies with a score of 1 to 3 were of low-quality and a score of 4 to 7 indicated high-quality.

### 2.4 Data extraction

The following data were extracted: authors, year of publication, mean age of treatment group and control group, the number of patients (treatment group/control group), diagnostic criteria, interventions, and duration of treatment.

The main evaluation indices were as follows:

(1) renal function indicators: 24-h U-Pro, Scr, BUN;
(2) serological indicators: ALB;
(3) Drug safety evaluation: number of patients with adverse event relative to the total number of patients.

The secondary evaluation indices were:

(1) Renal function indicator: Ccr (creatinine clearance);
(2) Blood lipid indicators: TG and CHOL.

### 2.5 Sensitivity analysis

Sensitivity analysis was performed for each variable by eliminating one study and recalculating the data of the remaining studies to determine the effect of the variable on the results. The absence of any major changes indicates stable results.^[11]^

### 2.6 Heterogeneity analysis

*I*^2^ was used to determine the heterogeneity of the included studies, with *P* < .05 indicating statistical significance.^[12]^ Fixed effects model was used for *I*^2^ < 50% and *P* ≥ .05, otherwise a random-effects model was used.

### 2.7 Subgroup analysis

The heterogeneity between studies was evaluated by the *I*^2^ index. The following subgroups were analyzed to identify the potential sources of heterogeneity: Nephrotic syndrome (NS is defined as proteinuria >3.5 g/d and serum albumin <30 g/L), glomerulonephritis (proteinuria <3.5 g/d and serum albumin >30 g/L), and other (no clear description of proteinuria or serum albumin) stages based on the clinical manifestation,^[13]^ and based on the TCM treatment focus and the severity of Qi deficiency and blood stasis syndrome,^[14]^ invigorate Qi (Qi deficiency > blood stasis), dispel blood stasis (blood stasis > Qi deficiency), or both (blood stasis = Qi deficiency).

### 2.8 Publishing bias

Begg test and funnel plot were used to determine publication bias with the State software. A roughly symmetrical funnel plot, or a Begg test with *P* > .05 indicated lack of publication bias.^[15]^

### 2.9 Statistical analysis

RevMan software v5.3 was used for meta-analysis and statistical analysis (The Cochrane Collaboration, Oxford, UK). Standard mean difference (SMD) and 95% confidence interval (CI) were calculated, and *P* < .05 was considered statistically significant.^[16]^

## 3 Results

### 3.1 Study selection and literature search

**Figure 1:**
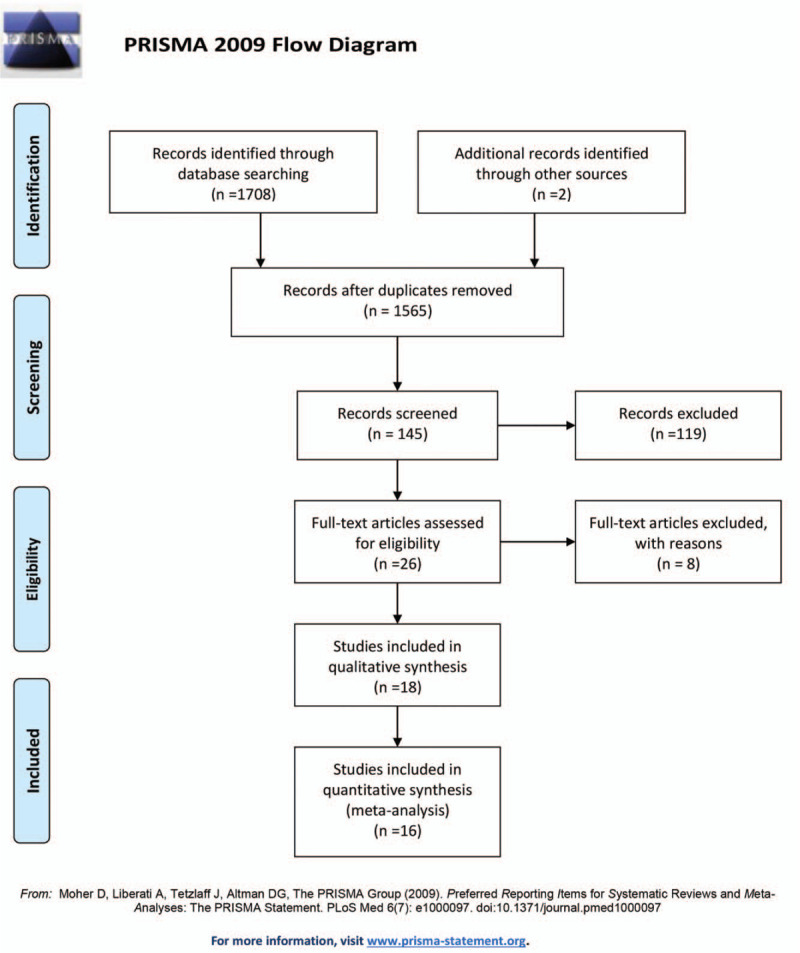
Flow diagram of the literature search and study selection.

A total of 1710 articles were retrieved, of which 1565 were excluded based on their titles and abstracts. After excluding 129 articles based on the criteria mentioned in the methods, 16 articles that met the inclusion criteria were finally selected for meta-analysis (Fig. 1). The RCTs are summarized in Table 1. As shown in Table 2, the highest Jadad score was 5, and the average score was 3.63.

**Table 1:** Characteristics of the RCTs included for the meta-analysis.

		Mean age		No. of patients			Intervention strategy		
First author	Year of publication	Treatment group	Control group	Treatment group	Control group	Diagnostic criteria^∗^	Treatment group	Control group	Duration
Xiao-Xia Cheng^[17]^	2001	34.27 ± 11.64	33.33 ± 11.05	30	15	A, B, E	Control + yishen tongluo decoction (6 Chinese herbs)	Pred, T_1_, CTX, ACEI	16 weeks
Xing-Cai He^[18]^	2007	42.73 ± 3.22	43.33 ± 3.45	50	50	A, B, C, E	Control + qingxue xiaobai decoction (12 Chinese herbs)	Pred, CTX	12 weeks
Cai-Feng Zhu^[19]^	2007	32.00	34.68	35	31	A, B	Control + (13 Chinese herbs)	ACEI/ARB + Fish oil + therapy for disease	24 weeks
Wen-Gang Guo^[20]^	2014	37.1	36.2	20	15	A, B, C, E	Control + (9 Chinese herbs)	Pred, CTX	24 weeks
Hai-Yan Lv^[21]^	2016	43.1 ± 3.0	42.1 ± 3.4	50	50	A, B, D, E	Control + self-designed peishen decoction (14 Chinese herbs)	GC+FK506	6 weeks
Zhi-Jie Dang^[22]^	2017	34.1 ± 1.2	35.3 ± 0.9	45	45	A, B, C, E	Control + (10 Chinese herbs)	Pred, CTX	24 weeks
Xu-dan Heng^[23]^	2017	42.1 ± 7.4	43.1 ± 6.7	42	42	A, B, D, E	Control + peishen decoction (9 Chinese herbs)	Hormones+FK506	48 weeks
Hui Feng^[24]^	2014	-	-	30	30	A, B, E	Control + (10 Chinese herbs)	Hormones + stomach protection + calcium supplement	12 weeks
Xin-wei Wang^[25]^	2010	52.25	50.5	22	20	A, B, C, E	Control + shenzong huoxue decoction (14 Chinese herbs)	Anticoagulant + lipid-lowering + immunosuppressant	12 weeks
Xiao-hua Yan^[26]^	2013	38.07 ± 11.03	34.17 ± 8.99	30	30	A, B, E	Control + pishen tongluo decoction (9 Chinese herbs)	ACEI	8 weeks
Jiang Hai^[27]^	2015	39.26 ± 3.14	40.28 ± 3.16	45	45	A, B, C, E	Control + shenzong huoxue decoction (12 Chinese herbs)	Anticoagulant + lipid-lowering + immunosuppressant + hormone	48 weeks
Yan Liu^[28]^	2012	−	−	30	30	A, B, C, E	Control + yiqi tongluo decoction (8 Chinese herbs)	ACEI/ARB +Anticoagulant + lipid-lowering	8 weeks
Qing-zhen Liu^[29]^	2016	56.4 ± 7.8	55.6 ± 8.3	30	30	A, B, E	Control +yiqi tongluo decoction (11 Chinese herbs)	Hormones+FK506	12 weeks
Qiong-li Yin^[30]^	2017	49.61 ± 3.11	49.06 ± 3.09	50	50	A, B, E	Control +qingshen jiedu decoction (10 Chinese herbs)	ACEI	12 weeks
Qiu-xia Wu^[31]^	2009	−	−	15	15	A, B, E	Control +zhengqing fengtongning decoction	Hormones +ACEI +CTX	36 weeks
Jia-liang Guan^[32]^	2009	33.67 ± 9.94	32.72 ± 10.21	30	30	A, B, E	Control +jiedu fushen tongyu decoction (13 Chinese herbs)	Pred	8 weeks

**Table 2:** Quality scores of the included randomized clinical trials.

Author	Year of publication	Randomized	Randomization hide	Blinding	Withdrawal and exit	Jadad scores
Xiao-Xia Cheng^[17]^	2001	1	1	1	0	3
Xing-Cai He^[18]^	2007	2	1	1	0	4
Cai-Feng Zhu^[19]^	2007	1	1	1	0	3
Wen-Gang Guo^[20]^	2014	2	1	1	0	4
Hai-Yan Lv^[21]^	2016	1	1	2	0	4
Zhi-Jie Dang^[22]^	2017	1	1	1	0	3
Xu-dan Heng^[23]^	2017	2	1	1	0	4
Hui Feng^[24]^	2014	1	1	1	0	3
Xin-wei Wang^[25]^	2010	2	1	1	0	4
Xiao-hua Yan^[26]^	2013	2	1	1	0	4
Jiang Hai^[27]^	2015	2	1	1	0	4
Yan Liu^[28]^	2012	2	1	1	0	4
Qing-zhen Liu^[29]^	2016	1	1	1	0	3
Qiong-li Yin^[30]^	2017	1	1	1	0	3
Qiu-xia Wu^[31]^	2009	1	1	1	0	3
Jia-liang Guan^[32]^	2009	2	1	1	1	5

### 3.2 Meta-analysis results

#### 3.2.1 24-h U-Pro level

Eleven RCTs^[19,20,22–26,28–30,32]^ compared the 24-h U-Pro levels in the treatment (374 patients) and control groups (368 patients). As shown in the forest plot in Fig. 2 , there was considerable heterogeneity across the studies (*P* = .0002, *I*^2^ = 70%). Nevertheless, the 24-h U-Pro was significantly lower in the treatment versus the control group (SMD: 0.91 g/24 h, 95% CI: 0.63–1.19, *P* < .00001), indicating that the combination of TCM+WM can effectively reduce proteinuria. We conducted a subgroup analysis based on the treatment focus and the clinical manifestation of GS. Treatments that replenish Qi, dispel blood stasis, and have other effects reduced the 24-h U-Pro (SMD: 1.16 g/24 h, 95% CI: 0.77–1.55, *P* < .00001; SMD: 0.75 g/24 h, 95% CI: 0.30–1.20, *P* = .001; SMD: 0.84 g/24 h, 95% CI: 0.03–1.65, *P* = .04, respectively). Further subgroup analysis based on the clinical manifestation of glomerulosclerosis showed that TCM+WM significantly reduced 24-h U-Pro in patients with glomerulonephritis and nephrotic syndrome (NS) (SMD: 1.70 g/24 h, 95% CI: 1.20–2.20, *P* < .00001; SMD: 1.14 g/24 h, 95% CI: 0.87–1.41, *P* < .00001). For other subtypes also, TCM+WM significantly reduced the 24-h U-Pro (SMD: 0.64 g/24 h, 95% CI: 0.32–0.95, *P* < .0001). Furthermore, the differences were statistically significant across subtypes.

**Figure 2:**
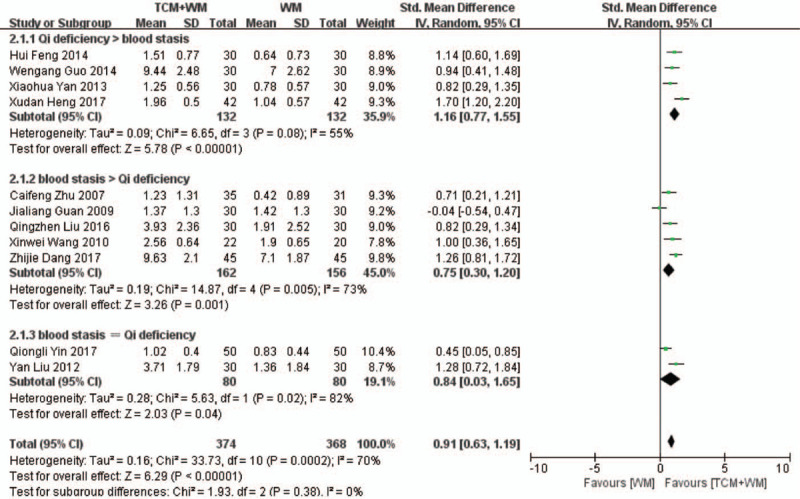
2.1 Subgroup analyses of 24-h U-Pro according to guiding ideology for TCM treatment of GS. (2.1.1) TCM treatment based on invigorating qi (Qi deficiency > blood stasis). (2.1.2) TCM treatment based on dispelling blood stasis (blood stasis > Qi deficiency). (2.1.3) Both (blood stasis = Qi deficiency). 2.2 Subgroup analyses of 24-h U-Pro according to clinical manifestation of GS. (2.2.1) Studies with glomerulonephritis participants. (2.2.2) Studies with nephrotic syndrome participants. (2.2.3) Studies with other participants. 24 h U-Pro = 24-hour urine protein quantity; GS = glomerulosclerosis; TCM = traditional Chinese medicine.

**Figure 2 (Continued):**
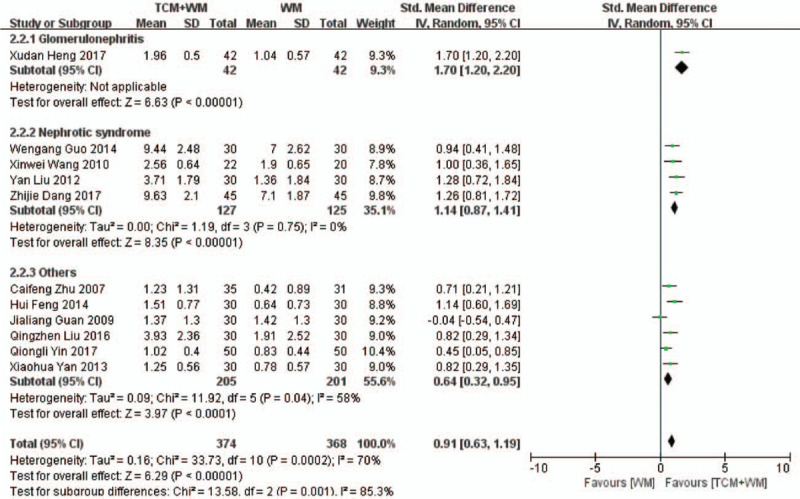
2.1 Subgroup analyses of 24-h U-Pro according to guiding ideology for TCM treatment of GS. (2.1.1) TCM treatment based on invigorating qi (Qi deficiency > blood stasis). (2.1.2) TCM treatment based on dispelling blood stasis (blood stasis > Qi deficiency). (2.1.3) Both (blood stasis = Qi deficiency). 2.2 Subgroup analyses of 24-h U-Pro according to clinical manifestation of GS. (2.2.1) Studies with glomerulonephritis participants. (2.2.2) Studies with nephrotic syndrome participants. (2.2.3) Studies with other participants. 24 h U-Pro = 24-hour urine protein quantity; GS = glomerulosclerosis; TCM = traditional Chinese medicine.

#### 3.2.2 Scr level

Twelve RCTs^[17–24,26,27,30,32]^ compared changes in Scr between the control and experimental groups. As shown in Fig. 3 , there was significant heterogeneity among the studies (*P* < .00001, *I*^2^ = 89%). TCM+WM reduced Scr levels to a greater extent compared with WM alone (SMD: 0.77 μmol/L, 95% CI: 0.35–1.18, *P* = .0003). The subgroup analysis showed that guiding ideology for TCM treatment of glomerulosclerosis based on replenishing Qi and both were beneficial to the TCM+WM group (SMD: 1.05 μmol/L, 95% CI: 0.43–1.68, *P* = .0010; SMD: 0.75 μmol/L, 95% CI: 0.38–1.12, *P* < .0001, respectively). Subgroup analysis further confirmed the superior effect of TCM+WM in patients with glomerulonephritis, NS, and other clinical diagnosis (SMD: 1.67 μmol/L, 95% CI: 0.71–2.62, *P* = .0006; SMD: 0.93 μmol/L, 95% CI: 0.11–1.76, *P* = .03; SMD: 0.34 μmol/L, 95% CI: 0.10–0.58, *P* = .006, respectively), and the difference was statistically significant among these groups.

**Figure 3:**
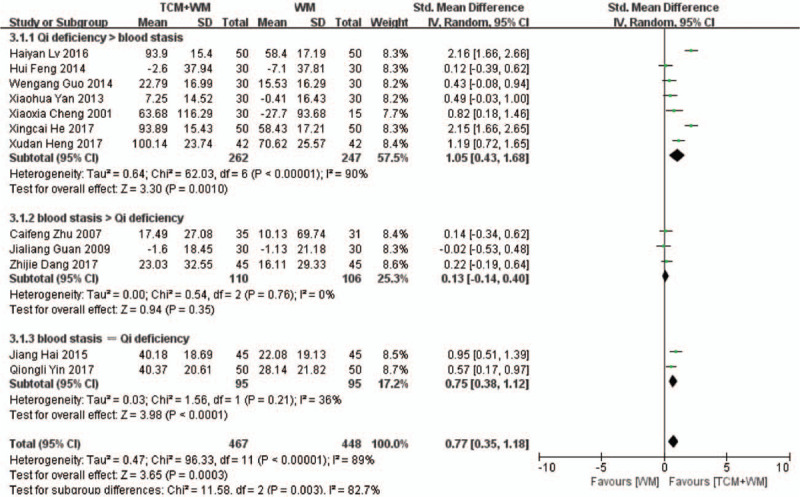
3.1 Subgroup analyses of Scr according to guiding ideology for TCM treatment of GS. (3.1.1) TCM treatment based on invigorating qi (Qi deficiency > blood stasis). (3.1.2) TCM treatment based on dispelling blood stasis (blood stasis > Qi deficiency). (3.1.3) Both (blood stasis = Qi deficiency). 3.2 Subgroup analyses of Scr according to clinical manifestation of GS. (3.2.1) Studies with glomerulonephritis participants. (3.2.2) Studies with nephrotic syndrome participants. (3.2.3) Studies with other participants. GS = glomerulosclerosis; Scr = serum creatinine; TCM = traditional Chinese medicine.

**Figure 3 (Continued):**
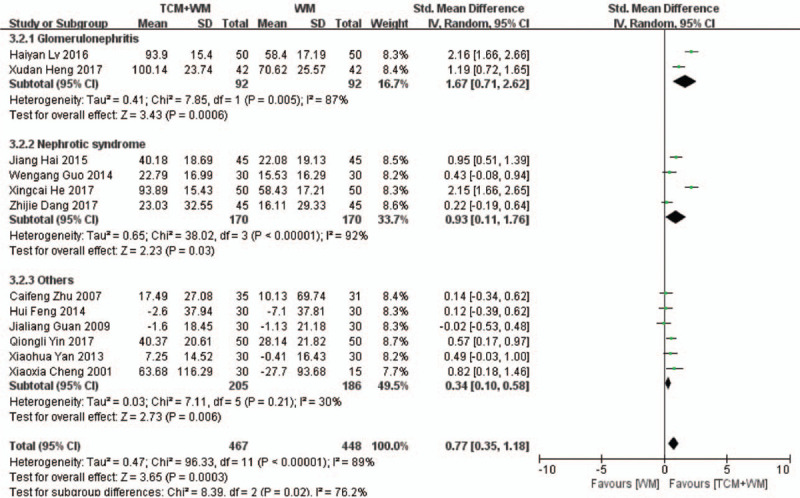
3.1 Subgroup analyses of Scr according to guiding ideology for TCM treatment of GS. (3.1.1) TCM treatment based on invigorating qi (Qi deficiency > blood stasis). (3.1.2) TCM treatment based on dispelling blood stasis (blood stasis > Qi deficiency). (3.1.3) Both (blood stasis = Qi deficiency). 3.2 Subgroup analyses of Scr according to clinical manifestation of GS. (3.2.1) Studies with glomerulonephritis participants. (3.2.2) Studies with nephrotic syndrome participants. (3.2.3) Studies with other participants. GS = glomerulosclerosis; Scr = serum creatinine; TCM = traditional Chinese medicine.

#### 3.2.3 BUN level

The BUN levels were compared in 11 RCTs,^[17–23,26,27,30–32]^ which showed high heterogeneity (*P* < .00001, *I*^2^ = 88%; Fig. 4 ). The BUN levels were significantly lower in the TCM+WM group (SMD: 0.83 mmol/L, 95% CI: 0.40–1.25, *P* = .0001), indicating that the combination of TCM+WM was more effective in lowering BUN levels. The subgroup analysis showed that the guiding ideology for TCM treatment of glomerulosclerosis based on replenishing Qi and both were beneficial to the TCM+WM group (SMD: 1.01 mmol/L, 95% CI: 0.29–1.72, *P* = .006; SMD: 1.12 mmol/L, 95% CI: 0.67–1.56, *P* < .00001, respectively). In addition, TCM+WM resulted in lower BUN in the glomerulonephritis and NS subgroups (SMD: 1.81 mmol/L, 95% CI: 0.22–3.40, *P* = .03; SMD: 0.94 mmol/L, 95% CI: 0.41–1.48, *P* = .0006, respectively).

**Figure 4:**
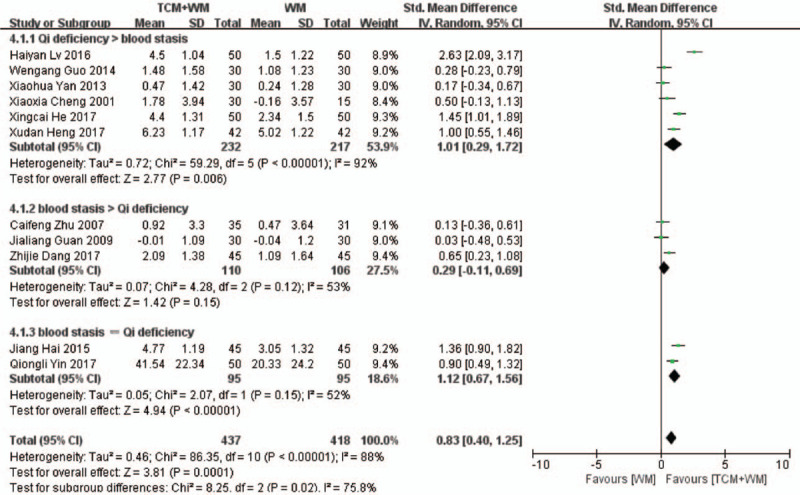
4.1 Subgroup analyses of BUN according to guiding ideology for TCM treatment of GS. (4.1.1) TCM treatment based on invigorating qi (Qi deficiency > blood stasis). (4.1.2) TCM treatment based on dispelling blood stasis (blood stasis > Qi deficiency). (4.1.3) Both (blood stasis = Qi deficiency). 4.2 Subgroup analyses of BUN according to clinical manifestation of GS. (4.2.1) Studies with glomerulonephritis participants. (4.2.2) Studies with nephrotic syndrome participants. (4.2.3) Studies with other participants. BUN = blood urea nitrogen; GS = glomerulosclerosis; TCM = traditional Chinese medicine.

**Figure 4 (Continued):**
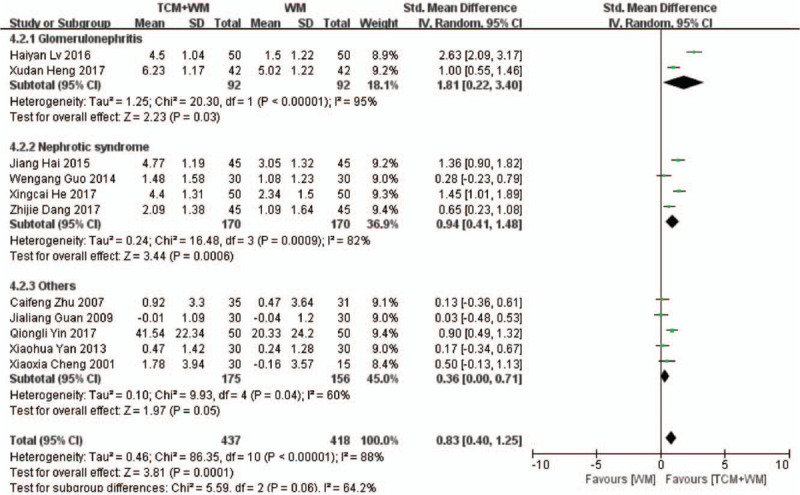
4.1 Subgroup analyses of BUN according to guiding ideology for TCM treatment of GS. (4.1.1) TCM treatment based on invigorating qi (Qi deficiency > blood stasis). (4.1.2) TCM treatment based on dispelling blood stasis (blood stasis > Qi deficiency). (4.1.3) Both (blood stasis = Qi deficiency). 4.2 Subgroup analyses of BUN according to clinical manifestation of GS. (4.2.1) Studies with glomerulonephritis participants. (4.2.2) Studies with nephrotic syndrome participants. (4.2.3) Studies with other participants. BUN = blood urea nitrogen; GS = glomerulosclerosis; TCM = traditional Chinese medicine.

#### 3.2.4 ALB level

Ten RCTs^[19–23,25,27,28,29,31,32]^ compared the levels of ALB between the control and treatment groups. As shown in Fig. 5 , there was considerable heterogeneity among the studies (*P* < .00001, *I*^2^ = 94%). The meta-analysis shows that the difference in ALB in the control and treatment groups was statistically significant (SMD: –1.49 g/L, 95% CI: –2.25 to –0.73, *P* = .0001), indicating that TCM+WM can control ALB levels more effectively. Subgroup analysis showed that the guiding ideology for TCM treatment of glomerulosclerosis based on replenishing Qi and both significantly improved ALB levels (SMD: –2.70 g/L, 95% CI: –4.28 to –1.12, *P* = .0008; SMD: –1.58 g/L, 95% CI: –2.08 to –1.08, *P* < .00001), and there was significant differences between across the treatment groups. Subgroup analysis also confirmed the beneficial effects of TCM+WM on 2 disease types (SMD: –2.37 g/L, 95%CI: –4.64 to –0.11, *P* = .04; SMD: –1.79 g/L, 95% CI: –2.92 to –0.66, *P* = .002)

**Figure 5:**
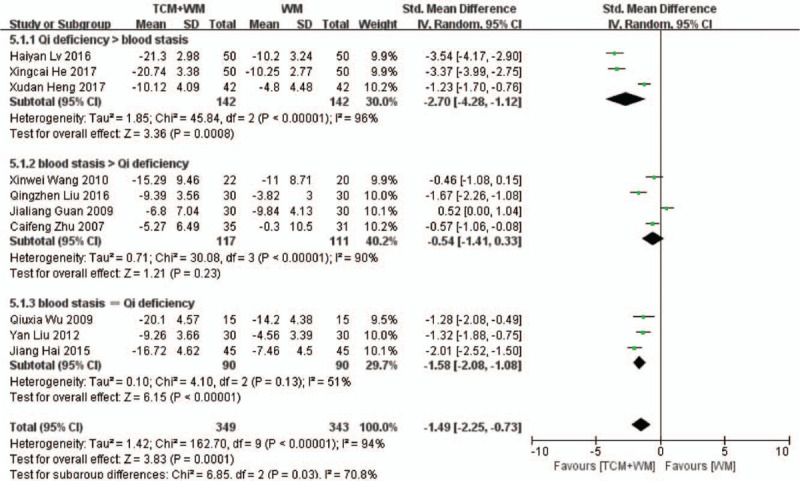
5.1 Subgroup analyses of ALB according to guiding ideology for TCM treatment of GS. (5.1.1) TCM treatment based on invigorating qi (Qi deficiency > blood stasis). (5.1.2) TCM treatment based on dispelling blood stasis (blood stasis > Qi deficiency). (5.1.3) Both (blood stasis = Qi deficiency). 5.2 Subgroup analyses of ALB according to clinical manifestation of GS. (5.2.1) Studies with glomerulonephritis participants. (5.2.2) Studies with nephrotic syndrome participants. (5.2.3) Studies with other participants. ALB = serum albumin; GS = glomerulosclerosis; TCM = traditional Chinese medicine.

**Figure 5 (Continued):**
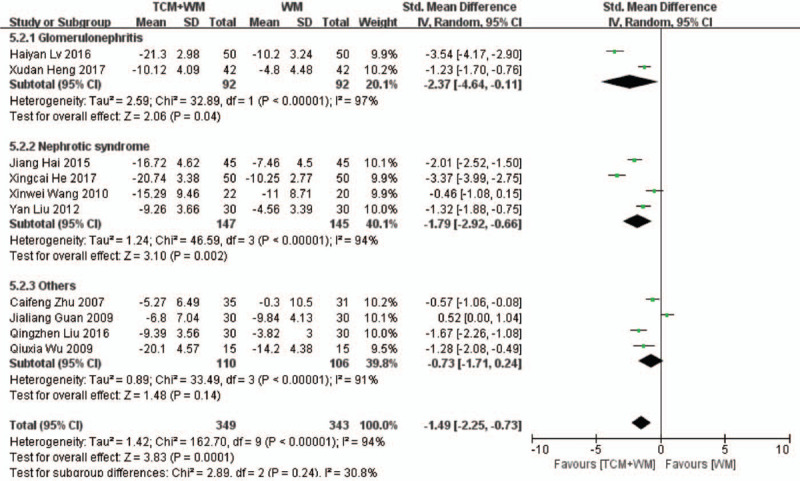
5.1 Subgroup analyses of ALB according to guiding ideology for TCM treatment of GS. (5.1.1) TCM treatment based on invigorating qi (Qi deficiency > blood stasis). (5.1.2) TCM treatment based on dispelling blood stasis (blood stasis > Qi deficiency). (5.1.3) Both (blood stasis = Qi deficiency). 5.2 Subgroup analyses of ALB according to clinical manifestation of GS. (5.2.1) Studies with glomerulonephritis participants. (5.2.2) Studies with nephrotic syndrome participants. (5.2.3) Studies with other participants. ALB = serum albumin; GS = glomerulosclerosis; TCM = traditional Chinese medicine.

#### 3.2.5 CHOL level

**Figure 6:**
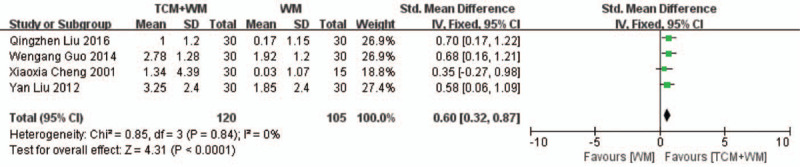
Comprehensive evaluation of CHOL after TCM+WM treatment. CHOL = cholesterol; TCM = traditional Chinese medicine; WM = Western medicine.

Four RCTs^[17,20,28,29]^ analyzed the level of CHOL before and after treatment, and were highly homogenous (*I*^2^ = 0%, *P* = .84). CHOL levels were significantly lower in the TCM+WM versus the WM group (SMD: 0.60 mmol/L, 95% CI: 0.32–0.87, *P* < .0001; Fig. 6).

#### 3.2.6 Ccr level

**Figure 7:**
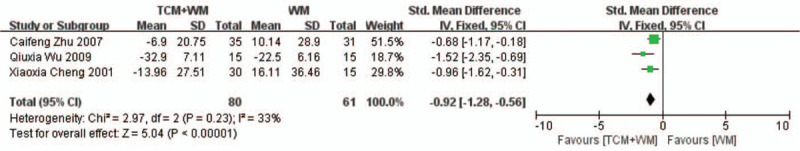
Comparison of Ccr level in TCM+WM and WM groups. Ccr = creatinine clearance; TCM = traditional Chinese medicine; WM = Western medicine.

Three RCTs^[17,19,31]^ compared the level of Ccr, and the level of heterogeneity was low (*I*^2^ = 33%, *P* = .23). Meta-analysis showed that compared with the control group, TCM+WM significantly increased the level of Ccr in patients with glomerulosclerosis (SMD = –0.92 mL/min, 95% CI: –1.28 to –0.56, *P* < .00001; Fig. 7).

#### 3.2.7 TG level

**Figure 8:**
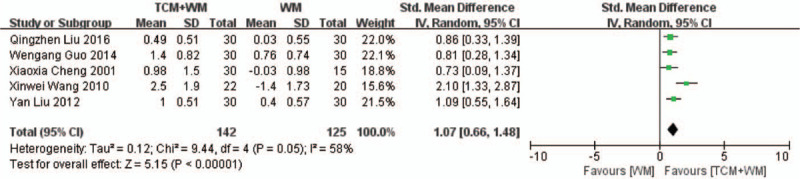
Summarizes the estimation of TG elevation after treatment with TCM+WM. TG = triglyceride; TCM = traditional Chinese medicine; WM = Western medicine.

Five RCTs^[17,20,25,28,29]^ compared the change in the level of TG, and showed remarkable heterogeneity (*P* = 0.05, *I*^2^ = 58%). TCM+WM showed a superior therapeutic effect on TG levels. (SMD: 1.07 mmol/L, 95% CI: 0.66–1.48, *P* < .00001; Fig. 8).

### 3.3 Adverse events

**Figure 9:**
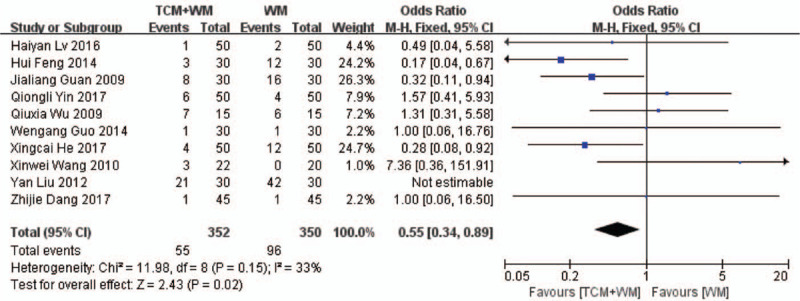
Forest plot of the safety of TCM+WM. TCM = traditional Chinese medicine; WM = Western medicine.

Thirteen RCTs evaluated the safety of TCM+WM^[17,18,20–22,24–26,28–32]^ in terms of gastrointestinal symptoms, infections, facial acne, liver damage, bone marrow suppression, thromboembolism, etc. Three studies reported absence of any adverse events during the treatment period,^[17,26,29]^ while 3 reported elevated blood sugar in 1 case each.^[20–22]^ In the 3 studies, there were 2 cases of “moon face” and 11 cases of respiratory tract and skin infections.^[18,24,32]^ In 1 study, patients experienced transient nausea and dizziness, which were mitigated with suitable drugs.^[25]^ Three studies reported 14 cases of gastrointestinal symptoms, 7 of Cushing-like manifestations, 6 of neuropsychiatric symptoms, 6 of insomnia, 3 of waist and knee soreness, 2 of liver damage, and 2 cases of bone marrow suppression.^[28,31,32]^ In 2 of these studies, some patients experienced multiple types of adverse events.^[28,32]^ One study recorded 5 cases with dizziness and fatigue, and 1 with decreased white blood cell counts.^[30]^ As shown in Fig. 9, the meta-analysis showed that the adverse events were significantly lower in the treatment group compared with the control group (Odds ratio (OR) = 0.55, 95% CI: 0.34–0.89, *Z* = 2.43, *P* = .02 < .05; Fig. 9).

### 3.4 Sensitivity analysis

Sensitivity analysis of 7 indicators (24 h U-Pro, Scr, BUN, ALB, TG, Ccr, and CHOL) did not show any significant change following elimination of single studies, indicating that the results were stable (Fig. 10 ).

**Figure 10:**
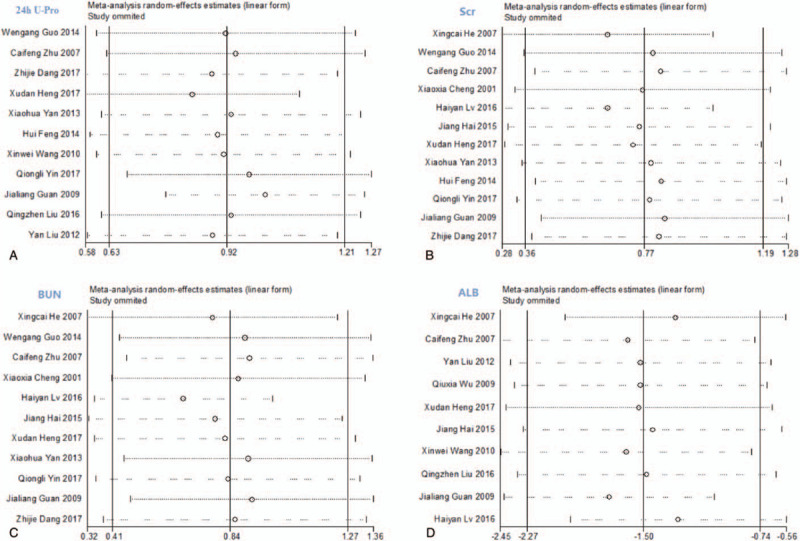
The results of sensitivity analysis.

**Figure 10 (Continued):**
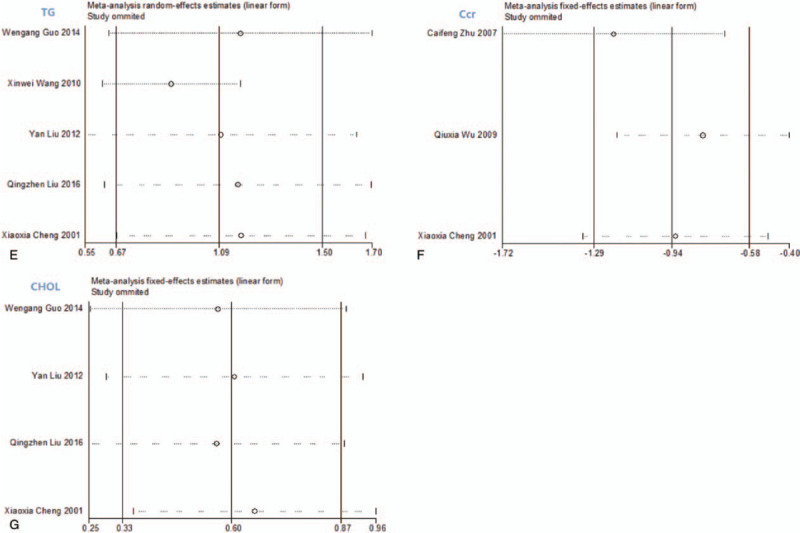
The results of sensitivity analysis.

### 3.5 The assessment of publication bias

The funnel plot of the 7 indicators (24 h U-Pro, Scr, BUN, ALB, TG, Ccr, and CHOL) did not show any significant publication bias in the meta-analysis (Fig. 11 ).

**Figure 11:**
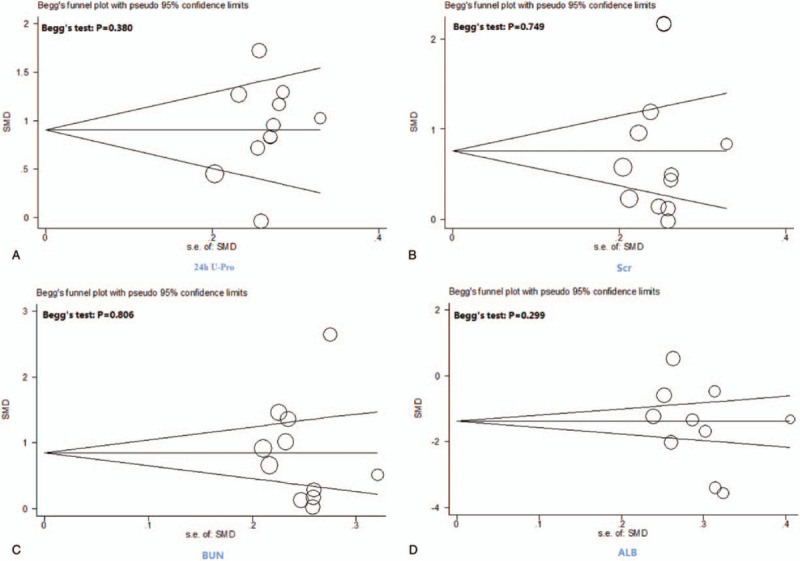
Funnel plots and Begg tests of publication bias.

**Figure 11 (Continued):**
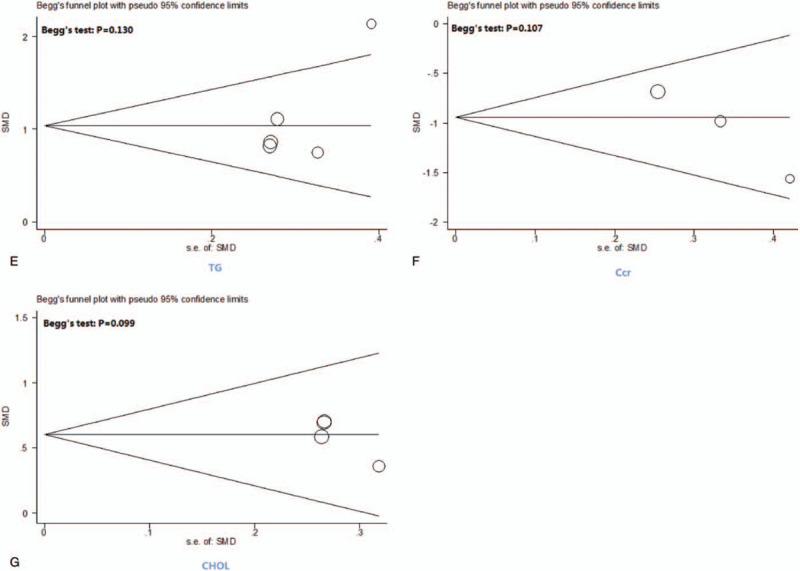
Funnel plots and Begg tests of publication bias.

## 4 Discussion

Glomerulosclerosis frequently progresses to end-stage renal disease, which is highly recalcitrant to treatment.^[33]^ Although hormone therapy can improve remission rate for 16 weeks, prolonged treatment may result in serious adverse reactions, such as blood pressure fluctuations, faster heart rate, decreased immune function, and secondary diabetes. In addition, immunosuppressants like FK506, cyclosporine A etc. are more expensive and cannot be prescribed often.^[31]^

Traditional Chinese medicine based on natural herbs has gained considerable attention in recent years due to the lower toxicity and side effects. However, the TCM formulations are not well defined and rarely validated by clinical studies. To this end, we performed a meta-analysis of 16 RCTs^[17–32]^ to compare the therapeutic effect of WM alone or in combination with TCM on 1082 patients with glomerulosclerosis. He et al^[18]^ applied self-made Qingxue Xiaobai decoction to mitigate the side effects caused by long-term hormone therapy, improve immunity, and reduce the recurrence of glomerulosclerosis, thereby delaying renal deterioration. Yan et al^[26]^ found that the Pishen Tongyu decoction can reduce renal protein levels and block connective tissue growth factor (CTGF) expression or inhibit its activity, thereby inhibiting renal fibrosis and delaying the progression of glomerulosclerosis. Hai^[27]^ used the Shenzong Huoxue decoction to increase the appetite of patients by restoring renal function, which increased protein intake and restored ALB and Hb levels. Modern pharmacological studies have demonstrated the reno-protective effects of TCM formulations.^[24]^ For example, rhubarb, Chuanxiong, and Tripterygium can relieve renal tubular hypermetabolism by inhibiting cell proliferation, reduce extracellular matrix accumulation, and resist platelet aggregation. In addition, Astragalus has a diuretic effect and can significantly reduce proteinuria. This meta-analysis showed that integrating TCM with conventional WM drugs can significantly improve renal function indices, improve treatment outcomes, and reduce recurrence. TCM+WM effectively reduced U-Pro, Scr, BUN, CHOL, and TG levels, and increased that of ALB and Ccr compared with WM alone.

The ideal meta-analysis should be able to include all high-quality, homogeneous studies. However, since it is practically difficult to include all studies, publication bias is unavoidable. In this study, funnel plots of the 24-h U-Pro, Scr, BUN, Ccr, TG, CHOL, and ALB showed incomplete symmetry, suggesting possible bias. The quality evaluation and risk bias analysis showed that the 16 included RCTs were very limited, and the amount of included studies was relatively less, which may lead to a result bias. Therefore, our conclusions need further validation through higher quality RCTs.

## 5 Conclusion

Integrated TCM+WM can significantly improve renal function, prognosis, and the quality of life of patients with glomerulosclerosis compared with WM alone, and should considered in clinical practice.

## Acknowledgments

The authors thank the researchers of the original studies included in this meta-analysis.

## Author contributions

**Conceptualization:** Yuetong Wang, Rong-qiang Zhang, Kai Qu.

**Data curation:** Yuetong Wang, Kai Qu.

**Formal analysis:** Yuetong Wang, Rong-qiang Zhang, Xian-cheng Li.

**Funding acquisition:** Kai Qu.

**Project administration:** Xiao-yong Yu, Kai Qu.

**Resources:** Yuetong Wang, Rong-qiang Zhang, Kai Qu.

**Software:** Yuetong Wang, Rong-qiang Zhang.

**Visualization:** Shu-fei Wang, Nan Zhang, Ya-feng Zhao, Yu Wang.

**Writing – original draft:** Yuetong Wang, Rong-qiang Zhang.

**Writing – review & editing:** Yuetong Wang, Rong-qiang Zhang, Kai Qu.
